# Household size explains successful malaria eradication

**DOI:** 10.1186/1475-2875-9-S2-O18

**Published:** 2010-10-20

**Authors:** Lena Hulden

**Affiliations:** 1 FI-00014 University of Helsinki, Finland

## 

Malaria is prevalent in 108 countries and has been eradicated from 80 countries. Malaria has only permanently disappeared in Palestine and Maldives because of complete eradication of vectors. Otherwise the explanation varies from changes in animal husbandry, switching *of Anopheles* species, draining of wetlands, use of dichlorodiphenyltrichloroethane (DDT), or changes in the climate.

Country data of malaria frequency was tested with a simple correlation test and a Chi Square test for household size, gross domestic product (GDP) per capita, population density and urbanization level. There was no correlation between malaria frequency and population density. Household size was the only factor with a specific threshold value when malaria disappeared, i.e. a household size lower than four members. This threshold value was also valid across all climate zones independently of 50-70 different vector species and 4(-5) different *Plasmodium* species.

Only seven countries made an apparent exception. Although they hade a low average household size, malaria was predominantly prevalent in areas or provinces where the average household size was higher than four. China was the only country for which the provincial statistics were too crude for proper analysis. Malaria frequency also has a high negative correlation with GDP and urbanization. GDP per capita does not as such explain malaria trends, but affects urbanization and household size. Increasing urbanization level improves housing conditions with an increasing share of multi-storey buildings, which reduces human contact with the vectors.

The importance of household size as the key explanatory variable for malaria prevalence implicates that land development, settlement planning and the planning of exploration of natural resources should be implemented in malaria eradication. Sudden and uncontrolled population movements into an area cause a rise of malaria cases. This can be seen in areas such as Rondonia and Mato Grosso in Brazil. Mosquito control alone cannot lead to malaria eradication, though it is essential for preventing epidemics. The extensive use of DDT in the 1940s and 1950s showed that in countries with a low household size eradication campaigns were successful but in countries with a household size above four members malaria returned with the DDT ban.

